# Is There a Movement Towards the Acceptance of Mindfulness in Medical Education? An Audit of Mindfulness Activity in UK Medical Schools

**DOI:** 10.15694/mep.2019.000084.1

**Published:** 2019-04-15

**Authors:** Lucy Harrison

**Affiliations:** 1 University of Oxford

**Keywords:** Mindfulness, Medical Education, Medical Students, Mental Health, Wellbeing

## Abstract

This article was migrated. The article was marked as recommended.

Within the UK, some medical schools have introduced secular mindfulness concepts to their students. However, knowledge of this development within academic medical institutions is largely anecdotal and not published. As such, this audit’s objective was to assess the scope of ‘Mindfulness Activities’ (MA) across UK medical schools.

In May 2018 a list of the current UK Medical Schools was retrieved from the Medical Schools Council website ( MSC, 2018) and the Heads of Faculty of each of the 38 UK medical schools were contacted and invited to take part, by completing a short survey. MA were categorised into three types of activity; ‘required’, ‘elective’ and ‘wellbeing’. ‘Required’ and ‘elective’ MA take place within curriculum time, whereas wellbeing MA was additional to the medical training.

Based on this audit, nearly 80% (30/38) medical schools have some form of MA accessible to their students.

This audit indicates that there is a relatively high level of MA within UK medical education. These findings, may indicate an acceptance of MT within the UK medical culture. More research is needed.

## Introduction

Within the UK, medical schools have already been introducing mindfulness to their students in various ways, from workshops, to lectures to complete courses; some have integrated it into their core curriculum. In an already packed medical curriculum some argue that there is insufficient space to include Mindfulness Training (MT). However, this position may be about to change in view of the wide-ranging reported benefits for medical students, doctors and patients.

It is well documented that medical students are at greater risk of stress, burnout, depression and addiction than the general population ( Dyrbye, Thomas and Shanfelt, 2006; Dyrbye *et al*., 2014; Jackson *et al*, 2016; Puthran, Zhang, Tam and Ho, 2016; Rotenstein *et al*., 2016). This is a serious, widespread concern because of the potential negative influence on clinical skills and patient care. Clinicians work in complex, emotionally demanding settings, where high-risk ethical and moral decisions are made. However, the embedding of approaches that promote self-care, self-awareness and compassion into the traditional model of medical education has been slow. Nevertheless, the focus on wellbeing is growing, with the intention of creating clinicians who are better equipped to take care of themselves and better resourced to serve their patients. Mindfulness Training (MT) is one such approach to self-care, and a number of medical schools already include components for their students. There are two main contexts in which MT is relevant to medical education: the first is as an intervention to promote wellbeing; the second is to promote the development of clinical skills.

Mindfulness is a universal skill; in its simplest form, is the ability to focus, non-judgementally on the present, whilst acknowledging but letting go of distracting influences ( Williams and Penman, 2014). Mindfulness has a growing scientific evidence base, which supports the psychological and physical benefits that arise through its practice. Mindfulness-Based Cognitive Therapy (MBCT) is an example of a Mindfulness-Based Intervention (MBI) which has been approved for use in the UK’s National Health Service since 2004 for the prevention of recurrent depression ( NICE, 2015). MBIs are secular manualised group-based intervention programs. They generally consist of 8 weekly 2-2.5-hour classes that carry approximately 15 participants. A key feature is the education in formal and informal mindfulness meditation practices to train both the attentional control component as well as the non-judgemental attitudinal aspects of mindfulness. Empirical research has demonstrated that MBCT significantly reduces relapse of depression in those with three or more previous episodes ( Chiesa and Serretti, 2011; Kuyken *et al*., 2016; Ma and Teasdale, 2004). MBIs are gaining a scientific foundation for use in a variety of other conditions including anxiety ( Hofmann, Sawyer, Witt and Oh, 2010), fibromyalgia (Grossman, Neimann, Schmidt and Walach, 2004), health anxiety ( Surawy, McManus, Muse and Williams, 2015), adults on the autistic spectrum ( Spek, Van Ham, Nadia and Nyklíček, 2013) and young people experiencing depression and anxiety ( Biegel, Brown, Shapiro and Schubert, 2009).

It is reasonable to comment that if healthcare is evidence-based, targeted wellbeing interventions to enhance the wellbeing and clinical performance of medical students should also be evidence-based. Drawing from the current available empirical data there is emerging evidence that MT could be a wellbeing intervention, and a high-level clinical skill. If MT could improve educational outcomes within medical training it could be an important addition to the medical school curricula. However, at present, there are only a number of small studies that indicate possible benefits of MT for medical students (See Supplement 1: Table Showing Review of Research Studies Relevant to Mindfulness and Medical Students), none of which are from the UK. As such, the full therapeutic and functional potential of MT within UK medical education is unknown and care must be taken when considering its implementation.

However, there are other areas of research, which are interesting and relevant. Within university students, MT can reduce stress ( Regehr, Glancy and Pitts, 2013), promote resilience ( Pidgeon *et al*., 2014), enhance wellbeing ( Galante *et al*., 2018) and improve memory and learning ( Ramsburg and Youmans, 2014). It can also improve cognitive flexibility and problem solving ( Greenberg, Reiner and Meiran, 2012). MT can enhance the wellbeing of clinicians, at the same time reducing burnout and increasing work engagement, and is also a highly promising intervention method for enhancing empathy ( Burton *et al*., 2017; Dean *et al*., 2017; Krasner *et al*., 2009; Lamothe *et al*., 2016; Shapiro, Schwartz and Bonner, 1998). MT is associated with greater communication skills, rapport and more satisfied patients ( Beach *et al*., 2013; Beckman *et al*., 2012). It also appears to reduce vicarious stress and career fatigue ( Breines and Chen, 2012)and foster self-compassion ( Boellinghaus, Jones and Hutton, 2012). There is also evidence that MT can reduce cognitive biases and clinical errors ( Sibinga and Wu, 2010; Thammasitboon and Cutrer, 2013).

In 2017, a special interest group was created called ‘Mindfulness in Medical Education’ (MiME). Their objective is to create a dialogue across medical schools, from which to share opportunities related to MT and medical education. Their work will be instrumental in guiding educators with respect to Mindfulness-based curriculum development.MiME is part of the Association for the Study of Medical Education (ASME). ASME’s mission is to meet the needs of teachers, trainers and learners in medical education and support research-informed, best practice across the continuum of medical education. Hence, an understanding of the current level of MA across UK medical schools is of relevance and importance to medical students, educators and clinicians.

## Methods

In May 2018 a list of the current UK Medical Schools was retrieved from the Medical Schools Council website ( MSC, 2018) and the Heads of Faculty of each of the 38 UK medical schools were contacted and invited to take part, by completing a short survey. This survey was reviewed by the Medical Sciences Interdivisional Ethics Committee at the University of Oxford who confirmed that it did not require ethical review. The Heads of Faculty were given 4 weeks to respond and, where necessary, a reminder was sent after 2 weeks. A website search was carried out for the medical schools that did not respond.

MA were categorised into three types of activity; ‘required’, ‘elective’ and ‘wellbeing’. ‘Required’ and ‘elective’ MA take place within curriculum time, whereas wellbeing MA was additional to the medical training.

## Results/Analysis

Twenty medical schools responded ( table 1). Eighteen did not respond; for this group a systematic website content search was carried out to assess MA related to education, wellbeing, and research ( table 2).

Nearly 80% (30/38) medical schools have some form of MA accessible to their students which is very encouraging. Based on this audit, the most common source of MA exists through wellbeing services (with 5 universities providing access to 8-week ‘wellbeing’ MBI courses). There are more examples of elective MA than required MA (with 2 medical schools offering an adapted MBI as an SSC). MA is in some way embedded as a required element into the core curriculum within 7 medical schools. Such embedding is wide-ranging, with the majority providing it early in training through wellbeing and resilience lectures comprising introductions to mindfulness research, concepts and practice (rather than an MBI format).

Three medical schools stand out because they have embedded a required (assessed) longitudinal, core curricular mindfulness program which stretches through the pre-clinical medical training. This course is based on a Health Enhancement Programme (HEP), which has been in use at Monash University since 2002, which will be discussed later.

Through website searches, I discovered that 3 of the medical schools that did not respond are within universities that offer Masters programmes in MBIs; they are also affiliated with mindfulness centres and the ‘The Mindfulness Network’. The UK Network for the Mindfulness-Based Teacher Training Organisations, which iscommitted to supporting and developing good practice and integrity in the delivery of MBIs by having strong collaborative relationships between member organisations. Defining, upholding and disseminating standards. Their websites indicated that 2 of them offer MBIs for wellbeing and one offers an elective SSC. There was no available information regarding required courses. However, I would I anticipate that they would be more committed to providing and/or teaching mindfulness in view of the level of engagement with mindfulness by the university more generally. Such association could provide a vehicle for dissemination of mindfulness-related research and a potential access point for future empirical evaluation.

**Table 1.** Information: Responsive Medical Schools (n=20) (Completed Questionnaires)

Type of Mindfulness Activity	Number of Medical Schools *that responded to audit (n=20/38)*	Additional Comments
Required	7/20	- 3 = Based on HEP program *assessed* - 4 = other (various formats/delivery including introduction to MT research, concepts, practice) *required but not assessed*
Elective (SSC)	11/20	- Length varies most 4 or 6 weeks - All assessed - 1 = MBI - 10 = other
Wellbeing *included those services that were available to any student within the university*	15/20	- 3 = access MBIs - 12= various (drop in or not stated) *Many offer links to online resources*
Any MA	18/20	= 90% of respondents
No MA	2/20	= 10% of respondents - Both of these medical schools are actively looking at SSC - 1 has a proposal to integrate mindfulness into the curriculum

**Table 2.** Information: Non-responsive Medical Schools (n=18) (Websites Search)

Type of Mindfulness Activity	Number of Universities *Website search* of *non-responders* *(n=18/38)*	Additional Comments
Required	No information available on website	- Unable to comment further on any of the 18 medical schools
Elective (SSC)	1	- 1= adapted SSC (6 weeks) - Lack of sufficient detail for the remaining 17 medical schools to comment further
Wellbeing *included those services that were available to any student within the university*	11	- 2 = access to MB - 9= drop in sessions via wellbeing services - Lack of sufficient detail for the remaining 6 medical schools to comment further
No information regarding mindfulness found on website	7	

**Figure 1.**
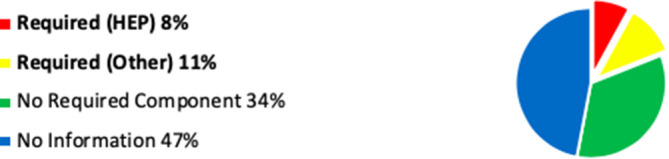
Chart Showing Assimilated (Questionnaire and Website) Data: ‘Required’ Mindfulness Activity

**Figure 2.**
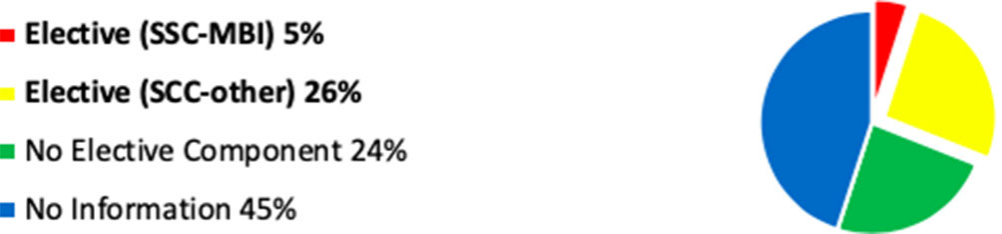
Chart Showing Assimilated Data: ‘Elective’ Mindfulness Activity

**Figure 3.**
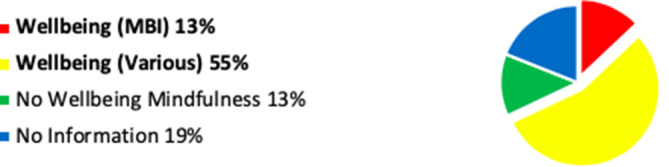
Chart Showing Assimilated Data: ‘Wellbeing’ Mindfulness

Whilst the website search information is not as accurate as the questionnaire response, this combined data has enabled further observation and comment, which I have illustrated in figures 1-3.

## Discussion

Whilst these results are not a full representation of the cohort of 38 medical schools, the high level of MA suggests that ‘mainstream’ academic medicine has been relatively receptive to mindfulness, at least as a means of voluntary involvement in self-care. However, this audit only gives a snapshot of MA within UK medical schools, it does not provide information about the level of integration within curricular and to my knowledge, there are no UK medical schools that have published empirical research into their programmes.Developing evidence-based MT aimed at the well medical student population, to support wellbeing and clinical performance, presents new and unique opportunities as well as challenges.

One interesting approach is the ‘required’, core-curricular Health Enhancement Programme (HEP), which is assessed and has been adopted by 3 UK medical schools. This was developed by Dr Craig Hassed of Monash University, Australia, where it has been in use since 2002. The HEP combines didactic teaching and experiential tutorials to explore the major ‘pillars of health’ necessary for wellbeing (stress release - through mindfulness, exercise, nutrition, connectedness, spirituality and environment). It devotes over 50% of its time and content to mindfulness. HEP integrates with biomedical sciences and clinical skills andforms a structure upon which educational, preventive and therapeutic approaches can be built.While there is no compulsion or requirement to practice mindfulness, students are encouraged to experiment with it in their own lives. Monash University was the first medical school, worldwide, to integrate MT into their core-curricular and the first to empirically support it ( Hassed, de Lisle, Sullivan and Pier, 2009).Student evaluation based on pre and post course questionnaires demonstrated significant improvements in several measures of self-perceived psychological wellbeing, this being directly related to the degree to which the students adopted mindfulness practice ( Hassed, de Lisle, Sullivan and Pier, 2009). However, limitations included absence of control, short length of follow up (6 weeks), self-report data, possible confounding factors (e.g. curriculum structure, delivery and adjustment to university life). Future research regarding long term outcomes and also data from the other medical schools using HEP would be helpful.Whilst Hassed and colleague’s study was not a ‘gold standard’ randomised controlled trial (RCT), it is now a ‘tried and tested’ program that has been successfully in use for at least 16 years. Indeed, the HEP model is being used in a number of medical schools worldwide (Australia (Monash, Deakin), UK (Leicester, Warwick and Aston), Canada (Montreal, McGill, Dalhousie), US (Harvard, UMass) New Zealand (Auckland), which also suggests that it is an adaptable model. Interestingly, these medical schools utilise the help and support of the developers of the HEP, who assist in making any relevant adaptations for individual medical schools. Whilst there is no available published outcome data for these medical schools, it is likely that they are evaluating their own courses.

There are several challenges to conducting and interpreting MT research. The RCT is the most scientifically rigorous method of hypothesis testing available, and is considered to provide the most reliable evidence on the effectiveness of interventions ( Akobeng, 2005). However, blinding cannot be implemented in RCTs of MT, which leads to a risk of bias. MT consists of, not just mindfulness meditation, but also psycho-education and yoga-like exercises, therefore it is difficult to pinpoint the mechanisms which contribute the most to the observed improvements. There is often cross-study variability in the content/format of the MT offered, making comparison and generalisation of findings difficult. Students attending mindfulness programmes as an elective may not be typical of the entire cohort. The self-reports of medical students are not proof of efficacy. Information regarding MT teaching fidelity, and the level of home practice that participants engaged with is often lacking from studies. Additionally, it is not always clear if the MT required extra time commitment or if it was an optional pathway within curricular time. Academic pressures may create a significant barrier to student participation in extracurricular stress reductions programs.

### Future Directions

I believe that this field of research could benefit from the findings of the Galante and colleagues ( 2018)study. Their promising research indicates that MT adapted for university students is an accessible, acceptable, feasible, and effective component of wider mental health strategies. Helpful future directions could be to trial elective MT for medical students, in various universities, across different socio-demographic groups. Within medical education, it would be beneficial to perform RCTs with robust methodologies to examine comparative efficacy; including large samples and comparison with other interventions to consider the variance and interdependence of influential factors (e.g. contact time and group dynamics). Pertinent long-term outcome data could usefully include measures for wellbeing, clinical performance and patient outcomes. The complexity of learning MT within medical training merits the use of a broad range of outcome measures to determine the correlation of stress reduction with clinical performance (e.g. biomarkers and neuroimaging). Qualitative research could complement quantitative research to explore both outcome and process variables and might lead to testable hypotheses and inform future research priorities. It would also be helpful to explore different methods of delivery (e.g. Online/DVDs) to support accessibility and to monitor for any adverse effects. The use of Galante and colleague’s ( 2018) standardised format for the MT intervention ‘Mindfulness Skills for Students’(MSS), could allow for replication and comparison studies. MSS is an adapted 8-week MBI course for university students. If such research supports elective MT then the next step could be to test an equivalent course in the context of a required course.

An ideal approach would be to provide a systematic review of the evidence and build an evidence base of MT efficacy within UK medical education. Individual medical schools could then devise their own evidence-based implementation plan, based upon robust clinical evidence from systematic research combined with students’ and teachers’ values and expectations. An appropriate opportunity for integrating MT could be during a reform of curricular, with self-care integrating with biomedical knowledge, clinical skills and assessment.

## Conclusion

This audit indicates that there is a relatively high level of MA within UK medical education.These findings, may indicate an acceptance of MT within the UK medical culture. Although several questions remain to be considered, the empirical research indicates that MT could play an important role in medical education and it is likely that the ever-growing evidence base for MT is changing perceptions of teachers, students and the medical profession more widely. However, an isolated MT intervention cannot fix a dysfunctional workplace or an unsustainable workload. MT will only realise its full potential when it is part of a well-designed organisational culture which takes medical student and clinician wellbeing seriously. A culture which also extols the fundamental principles of humanistic patient care.

## Take Home Messages

• Mindfulness Activity within the UK medical schools is relatively high, but this is wide ranging
• The concerns related to medical student wellbeing are significant
• Evidence is growing that mindfulness could aid medical student wellbeing and clinical skills
• More research is necessary to determine the usefulness of Mindfulness Training within medical education

## Notes On Contributors

Dr Lucy Harrison is a General Practitioner and Mindfulness-Based Cognitive Therapy Teacher who works in Newcastle Upon Tyne (United Kingdom). She completed a Masters in Mindfulness-Based Cognitive Therapy at the University of Oxford 2018. Her Mindfulness work centres around medical practitioners. ORCID: https://orcid.org/0000-0001-6303-4951
