# Supplementary material for: Is There a Movement Towards the Acceptance of Mindfulness in Medical Education? An Audit of Mindfulness Activity in UK Medical Schools
Source: MedEdPublish (2016). 2019 Apr 15;8:84. [Version 1] doi: 10.15694/mep.2019.000084.1 (PMC10712599; doi:10.15694/mep.2019.000084.1)
Supplement: Peer review history

# Reviewer report: Reviewer response for version 1

**DOI:** 10.21956/mep.19820.r29851

**Published:** 2019-07-30

**Authors:** Jayne Garner

**Affiliations:** 1 university of liverpool

This review has been migrated. The reviewer awarded 5 stars out of 5 This is a very timely and useful audit. The author presents a good range of background information and provides useful context regarding mindfulness in medical education. More empirical evidence of delivery of mindfulness activities and students reactions and experiences of these would be very welcome. Thank you for this valuable contribution.

Reviewer Expertise:

NA

No decision status is available

# Reviewer report: Reviewer response for version 1

**DOI:** 10.21956/mep.19820.r29852

**Published:** 2019-07-12

**Authors:** Trevor Gibbs

**Affiliations:** 1 AMEE

This review has been migrated. The reviewer awarded 4 stars out of 5 At a time when we are experiencing psychological issues with undergraduate students in our programmes, this paper is a very useful addition to our thinking on the subject of mindfulness and wellbeing.Although the paper ended with a call for more research, I did appreciite the authors approach to future directions and value the references given, which are incredibly helpful to all those seeking more insight into the subjectA very helpful paper for all those dealing with the struggling student

Reviewer Expertise:

NA

No decision status is available

# Reviewer report: Reviewer response for version 1

**DOI:** 10.21956/mep.19820.r29855

**Published:** 2019-04-25

**Authors:** Catherine McParland

**Affiliations:** 1 Nettleham medical practice

This review has been migrated. The reviewer awarded 5 stars out of 5 An excellent relevant audit!

Reviewer Expertise:

NA

No decision status is available

# Reviewer report: Reviewer response for version 1

**DOI:** 10.21956/mep.19820.r29854

**Published:** 2019-04-20

**Authors:** sathyanarayanan varadarajan

**Affiliations:** 1 SRM Medical College, SRM University

This review has been migrated. The reviewer awarded 4 stars out of 5 This innovative research article is about An Audit of Mindfulness Activity in UK Medical Schools. This audit’s objective was to assess the scope of ‘Mindfulness Activities’ (MA) across UK medical schools. This survey was done by contacting and inviting the Heads of Faculty of each of the 38 UK medical schools to take part, by completing a short survey. This audit indicates that there is a relatively high level of Mindfulness Activities within UK medical education and indicates an acceptance of Mindfulness Training within the UK medical culture. Although several questions remain to be considered, the empirical research indicates that Mindfulness Training could play an important role in medical education.This article will be helpful to all the medical educators who are interested in introducing Mindfulness Activities among medical students to tackle the risk of stress, burnout, depression and addiction. It’s high time to consider Mindfulness Training in Medical Education, which can create positive well being not only among medical students but also in medical teachers and improve clinical skills as well as patient care.

Reviewer Expertise:

NA

No decision status is available

# Reviewer report: Reviewer response for version 1

**DOI:** 10.21956/mep.19820.r29853

**Published:** 2019-04-17

**Authors:** Felix Silwimba

**Affiliations:** 1 University of Lusaka

This review has been migrated. The reviewer awarded 5 stars out of 5 This medical education audit report has met by long held thoughts on gaps I have experienced in my medical education way back in the late 80s and early 90s. I have always believed I have sought of suffered from post-traumatic stress disorder and I see it in my colleagues too. I recommend such inclusion in medical education to be compulsory in all medical school’s world over. I agree with the recommendation and the need to care for the careers.

Reviewer Expertise:

NA

No decision status is available
